# IgG, IgM, and IgA Antinuclear Antibodies in Discoid and Systemic Lupus Erythematosus Patients

**DOI:** 10.1155/2014/171028

**Published:** 2014-03-05

**Authors:** Sheridan A. Jost, Lin-Chiang Tseng, Loderick A. Matthews, Rebecca Vasquez, Song Zhang, Kim B. Yancey, Benjamin F. Chong

**Affiliations:** ^1^Department of Dermatology, UT Southwestern Medical Center, 5323 Harry Hines Boulevard., Dallas, TX 75390-9069, USA; ^2^Department of Clinical Sciences, UT Southwestern Medical Center, 5323 Harry Hines Boulevard., Dallas, TX 75390-9066, USA

## Abstract

IgG antinuclear antibodies (ANAs) are elevated in patients with systemic lupus erythematosus (SLE) compared with patients with discoid lupus erythematosus (DLE). To provide an expanded immunologic view of circulating ANAs in lupus patients, we compared the expressions of IgG, IgM, and IgA ANAs in DLE and SLE patients. In this cross-sectional study, sera from age-, gender-, and ethnic-matched SLE (*N* = 35), DLE (*N* = 23), and normal patients (*N* = 22) were tested for IgG, IgM, and IgA ANAs using enzyme-linked immunosorbent assays (ELISAs) and indirect immunofluorescence (IIF) with monkey esophagus as substrate. ELISAs showed elevated levels of IgG ANA, IgM ANA, and IgG/IgM ANA ratios in SLE patients compared with DLE and normal patients. IgA ANA expression was higher in SLE and DLE patients versus normal patients. IIF studies showed higher percentages of patients positive for IgG, IgM, and IgA ANAs in the SLE group. Higher IgG/IgM ANA ratios in SLE than DLE show enhanced class-switching and a more sustained humoral response in SLE. They also suggest a potential connection of IgM ANAs with disease containment.

## 1. Introduction

Discoid lupus erythematosus (DLE) and systemic lupus erythematosus (SLE) may or may not coexist, with DLE occurring in 20% of SLE patients [1] and 17% progressing to SLE [2]. Further distinctions have been made between the two diseases through observations of circulating IgG autoantibody levels. Previous studies have shown that IgG antinuclear antibodies (ANAs) are higher in SLE patients versus DLE patients [3, 4]. However, it is unknown whether levels of IgM or IgA ANAs can also be distinguished between DLE and SLE patients. To better understand immunologic relationships between DLE and SLE, we sought to compare the expressions of IgG, IgM, and IgA ANAs in patients with DLE and SLE by enzyme-linked immunosorbent assays (ELISAs) and indirect immunofluorescence (IIF). We hypothesized that the ANA levels for all three isotypes would be the highest in SLE patients, followed by DLE and normal patients.

## 2. Materials and Methods

### 2.1. Patients

Patients were recruited at Outpatient Dermatology and Rheumatology Clinics at the University of Texas Southwestern (UTSW) Medical Center from July 2003 to January 2011. Those giving informed consent to the study were enrolled into either the UTSW Cutaneous Lupus Registry or Dallas Regional Autoimmune Disease Registry. The study was approved by the UTSW Institutional Review Board and was performed according to the ethical standards established by the Declaration of Helsinki. Patients were divided into three age-, gender-, and ethnic-matched groups: SLE, DLE, and normal. SLE patients fulfilled at least four of the American College of Rheumatology (ACR) SLE diagnostic criteria [5], while DLE patients had a DLE diagnosis based on clinicopathologic correlation and less than four ACR SLE criteria. Normal controls were excluded if they had histories of autoimmune diseases. Demographics, medical history, and clinical data were collected for each patient. In addition, cutaneous and systemic disease activity for each DLE and SLE patient was measured by Cutaneous Lupus Erythematosus Area and Severity Index (CLASI) and Systemic Lupus Erythematosus Disease Activity Index (SLEDAI), respectively.

### 2.2. ELISAs

ELISAs were performed to measure IgG, IgM, and IgA ANAs, using commercially available ELISA kits (INOVA Diagnostics, Inc., San Diego, CA). ELISAs for IgG were ran according to the manufacturers' instructions, while the ELISA protocols for IgM and IgA ANA incorporated horseradish peroxidase-conjugated goat anti-human IgM (1 : 4,000 dilution) or IgA (1 : 5,000 dilution) second-step antibodies (Jackson ImmunoResearch Laboratories Inc., West Grove, PA). OD_450_ (optical density at 450 nm) values for IgM and IgA were obtained, and concentrations of IgG ANAs were calculated by extrapolating OD_450_ values to a standard curve.

### 2.3. Indirect Immunofluorescence

Six *μ*m cryosections of monkey esophagus tissue (Scimedx, Denville, NJ) were incubated with patient sera (1 : 20 dilution) at room temperature for 30 minutes in a humidified chamber, followed by three five-minute washes with 1X PBS. Fluorescein isothiocyanate-conjugated goat anti-human IgG (1 : 80 dilution), IgM (1 : 40 dilution), or IgA (1 : 80 dilution) (Invitrogen, Carlsbad, CA) was incubated on tissue cryosections and washed in the same manner, covered with a coverslip, and read by two blinded investigators (Benjamin F. Chong, Kim B. Yancey).

### 2.4. Statistical Analysis

Sample size was not calculated since this was a pilot study. We compared patient characteristics using Student's *t*-test or one-way analysis of variance (ANOVA) for continuous variables and Fisher's exact test or chi-squared test for categorical variables. For ELISA values, we used the Kruskal-Wallis test and Dunn's multiple comparisons post hoc test. Percentages of positive IIF results were compared using Fisher's exact test or chi-squared test. *P* < 0.05 was declared statistically significant.

## 3. Results and Discussion

### 3.1. ELISAs Show That SLE Patients Have the Highest IgG, IgM, and IgA Levels and IgG/IgM Ratios versus DLE and Normal Patients

**Figure 1:**
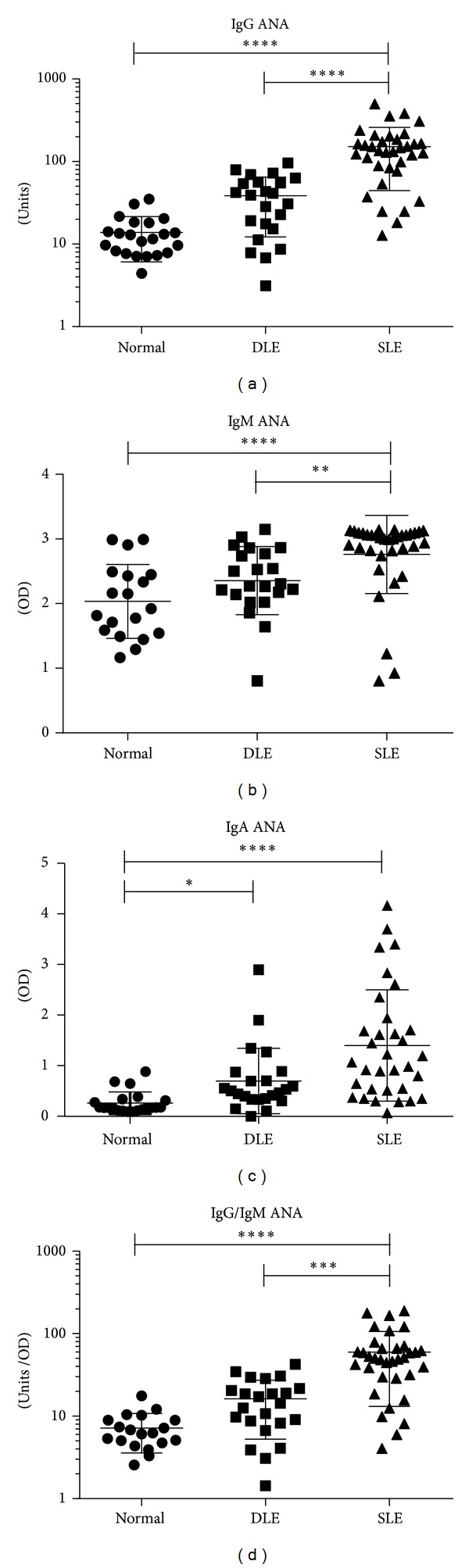
ELISAs showed significant differences in ANAs in DLE and SLE patients. ((a)–(d)) Levels of ANAs for IgG (a), IgM (b), and IgA (c), as well as the ratio of IgG/IgM ANAs (d), were measured in the sera of normal, DLE, and SLE patients. There are missing IgM ANA data for three normal samples and missing IgA ANA data for one normal sample due to insufficient quantities of sera. Kruskal-Wallis test and Dunn's post hoc test for multiple comparisons were performed for all analyses. *P ≤ 0.05, **P ≤ 0.01, ***P ≤ 0.001, and ****P ≤ 0.0001.

The demographics and clinical data from SLE (*N* = 35), DLE (*N* = 23), and normal (*N* = 22) patients were summarized in Table 1. SLE sera had higher IgG ANA expression (151.60 ± 107.00 units) compared with DLE (38.55 ± 26.35 units) and normal (13.83 ± 7.75 units) sera (*P* < 0.0001) (Figure 1(a)). Following a similar trend, SLE sera had increased IgM ANA expression (2.76 ± 0.60 OD) compared with DLE (2.36 ± 0.53 OD) and normal (2.04 ± 0.57 OD) sera (*P* < 0.0001) (Figure 1(b)). SLE (1.38 ± 1.09 OD) and DLE (0.69 ± 0.64 OD) sera contained higher IgA ANAs compared with normal (0.26 ± 0.21 OD) sera (*P* < 0.0001) (Figure 1(c)). None of these isotypes were exclusively elevated in any of these groups. Lastly, SLE sera had the highest ratio of IgG/IgM ANA (59.76 ± 46.64 units/OD) compared with DLE (16.27 ± 11.02 units/OD) and normal (7.18 ± 3.61 units/OD) sera (*P* < 0.0001) (Figure 1(d)).

### 3.2. IIF Studies Demonstrated That SLE Patients Had the Highest Rates of Positive IgG, IgM, and IgA ANA Staining

**Figure 2:**
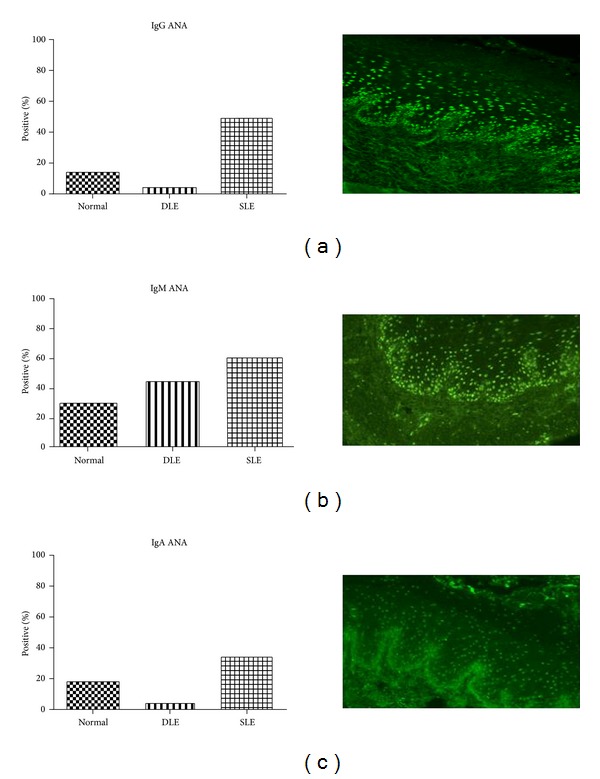
Higher percentages of positive ANAs were found in the SLE patient group by IIF. ((a)–(c)) Percentages of patients positive for IgG ANAs (a), IgM ANAs (b), and IgA ANAs (c) were calculated for normal, DLE, and SLE patient groups. There are missing IgM ANA data for three SLE samples due to insufficient quantities of sera. Examples of positive signals of IgG (a), IgM (b), and IgA (c) from SLE patients are shown. Objective: 200x.

Positive IgG staining against epithelial nuclei was seen in 17/35 (49%) SLE, 1/23 (4%) DLE, and 3/22 (14%) normal patients (*P* = 0.0002) (Figure 2(a)). IgM ANAs were present in 17/32 (53%) SLE, 9/23 (39%) DLE, and 6/22 (27%) normal patients (*P* = 0.16) (Figure 2(b)); 12/35 (34%) SLE, 1/23 (4%) DLE, and 4/22 (18%) normal patients exhibited positive IgA ANAs (*P* = 0.01) (Figure 2(c)). Most patients from all groups did not have IgG, IgM, or IgA staining against plasma membranes or basement membranes (data not shown).

### 3.3. Discussion

The ELISA and IIF results indicate that IgG and IgM ANAs are higher in SLE patients compared with DLE and normal patients. Thus, the trend of having greater amounts of circulating ANAs in SLE than DLE applies not only to IgG but also IgM. These differences both reflect the dichotomy between the systemic and skin-limited natures of SLE and DLE, respectively. As one of the ACR SLE diagnostic criteria [5], IgG ANAs target a variety of nuclear antigens such as double-stranded DNA (dsDNA), which are intimately involved in SLE pathogenesis. IgG anti-dsDNA antibodies injected into wild-type Balb-c mice and lupus-prone NZBxNZW F1 mice can induce and accelerate nephritis, respectively [6]. These antibodies can form immune complexes in circulation or bind to DNA exposed by glomeruli [7]. Subsequent events including complement activation [8], production of inflammatory mediators such as cytokines and chemokines, and activation of Fc*γ*R on phagocytes ultimately lead to tissue damage [9]. Less is known about IgM ANAs in SLE. Potential antigen targets of these antibodies include single-stranded DNA and dsDNA [10, 11]. Interestingly, IgM anti-dsDNA antibodies negatively correlate with presence of lupus nephritis in SLE patients [12]. Onset of nephritis was also delayed in NZBxNZW F1 mice injected with IgM anti-dsDNA antibodies [13].

We also found that the ratios of IgG/IgM ANAs were the highest in SLE patients. This implies an amplification of IgM to IgG class-switching and a more robust humoral response in SLE [14]. Moreover, lower IgG/IgM ANA ratios in DLE patients support IgM ANAs being associated with but not necessarily causative of disease containment [15]. Although IgM levels were higher in SLE than DLE patients, the increased ratio of IgG/IgM in SLE patients still hints at a protective effect of IgM. A similar phenomenon has been noted in SLE patients without lupus nephritis. They were found to have lower IgG/IgM ratios of anti-dsDNA antibodies compared with SLE patients with lupus nephritis [16]. It has been postulated that these IgM autoantibodies could decrease IgG autoantibody production by autoreactive B cells, diminish dendritic cell activation, or act as competitive inhibitors with their IgG counterparts by binding to the same circulating nuclear antigens [12, 17, 18]. These mechanisms may be potentially important in preventing systemic spread in DLE patients.

IgA ANAs were elevated in both DLE and SLE patients in the ELISA data. Moreover, IgA was the only immunoglobulin ANA isotype that was differentially expressed between normal and DLE patients. IgA deposits have been detected in the dermal-epidermal junction through direct immunofluorescence in 19/50 (38%) patients with DLE [19]. In MRL-lpr mice, which develop cutaneous lupus-like lesions, anti-desmoglein 3 IgA correlated with skin disease activity. Because the rise in anti-desmoglein 3 IgA was associated with mast cell infiltration in skin, it was postulated that anti-desmoglein 3 IgA could promote abnormalities in mast cell formation [20]. IgA deposits in skin have also been shown to drive the infiltration of neutrophils in various cutaneous autoimmune diseases such as linear IgA disease [21]. Neutrophils can aggregate in the dermal-epidermal junction in the skin of cutaneous lupus patients [22]. Upon exposure to anti-ribonucleoprotein antibodies, these neutrophils release extracellular traps, which contain dsDNA and other proteins that ultimately stimulate type I interferon production by plasmacytoid dendritic cells [23].

IIF studies on monkey esophagus rather than Hep2 cells were pursued so that we could detect IgG, IgM, and IgA binding against nuclei and other elements of cells in stratified squamous epithelium. Currently, no known antibody of any isotype is distinctly elevated in DLE patients. Desmoglein 3 was previously identified as a tissue-specific antigen in MRL-lpr mice [20]. However, DLE and SLE patients showed no significant IgG, IgM, or IgA against plasma and basement membranes, and the ELISA results showed no differences in anti-desmoglein-1 and -3 IgG between the two groups (data not shown).

Limitations include small sample size and selection bias, which was minimized by selecting age-, gender-, and ethnic-matched patients for each group. While IIF findings mostly mirrored the ELISA results, differences are likely due to the decreased sensitivity of detecting ANAs using monkey esophagus as substrate. Future ELISA and indirect immunofluorescence studies with Hep2 cells examining IgG, IgM, and IgA ANAs in a larger population of DLE and SLE patients could be performed to assess their diagnostic significance.

## 4. Conclusions

In this study, we have shown the differential expression of IgG, IgM, and IgA ANAs in DLE and SLE patients, providing a global picture of multiple isotypes of ANAs in these lupus subtypes. Decreased IgG/IgM ratios in DLE versus SLE imply amplified IgM to IgG class-switching in SLE and an association of IgM ANAs with prevention of disease spread. Increased IgA ANAs in DLE patients versus normal controls may suggest IgA having some involvement in the etiology of DLE.

## Figures and Tables

**Table 1:** Patient characteristics.

	Normal	DLE	SLE^§^	*P* value*
*N*	22	23	35	—
Age at visit, yr (SD)	43 (11)	43 (11)	42 (12)	0.93
Gender (M/F)	3/19	3/20	3/32	0.70
Ethnicity, *N* (%)				
Caucasian	6 (27)	6 (26)	6 (17)	0.60
African American	14 (64)	16 (70)	21 (60)	0.76
Hispanic	2 (9)	1 (4)	8 (23)	0.12
CLASI activity score, mean (SD)	N/A	7 (7)	10 (8)^†^	0.27
CLASI damage score, mean (SD)	N/A	8 (5)	11 (8)^†^	0.13
SLEDAI score, mean (SD)	N/A	1 (2)	3 (3)^‡^	0.03
Disease duration, mean (SD)	N/A	8 (9)	8 (10)^*£*^	0.88
Lupus medications at study visit, *N* (%)				
Topical/intralesional corticosteroids	N/A	9 (39)	10 (29)	0.40
Hydroxychloroquine	N/A	14 (61)	17 (49)	0.36
Chloroquine	N/A	3 (13)	1 (3)	0.29
Quinacrine	N/A	4 (17)	2 (16)	0.20
Methotrexate	N/A	2 (9)	1 (3)	0.56
Prednisone	N/A	0 (0)	19 (54)	<0.0001
Mycophenolate mofetil	N/A	1 (4)	10 (29)	0.04
Efalizumab	N/A	0 (0)	1 (3)	1.00
Leflunomide	N/A	0 (0)	1 (3)	1.00
Cyclophosphamide	N/A	0 (0)	1 (3)	1.00
None	N/A	6 (26)	3 (9)	0.13
SLE criteria, *N* (%)				
Malar rash	N/A	1 (4)	5 (14)	0.39
Discoid rash	N/A	23 (100)	17 (49)	<0.0001
Photosensitivity	N/A	15 (65)	17 (49)	0.21
Oral ulcers	N/A	3 (13)	12 (34)	0.12
Arthritis	N/A	2 (9)	19 (54)	0.0006
Serositis	N/A	0 (0)	11 (31)	0.002
Renal disorder	N/A	0 (0)	18 (51)	<0.0001
Neurological disorder	N/A	0 (0)	1 (3)	1.00
Hematological disorder	N/A	3 (13)	28 (80)	<0.0001
Positive ANA	N/A	8 (35)	34 (97)	<0.0001
Immunological disorder	N/A	0 (0)	32 (91)	<0.0001

^§^1 SLE patient met three criteria (renal disorder, positive ANA, and immunological disorder).

*P values for 2-group comparisons were calculated using Student's *t*-test for continuous variables and Fisher's exact test or chi-squared test for categorical variables, while *P* values for 3-group comparisons were calculated using one-way ANOVA for continuous variables and Fisher's exact test or chi-squared test for categorical variables.

^†^CLASI activity and damage scores were calculated for 16 SLE patients with discoid lupus.

^‡^SLEDAI scores were not calculated for eight SLE patients.

^£^Disease duration was not available for three SLE patients.

ANOVA: analysis of variance; CLASI: Cutaneous Lupus Disease Area and Severity Index; DLE: discoid lupus erythematosus; SLE: systemic lupus erythematosus; SLEDAI: Systemic Lupus Erythematosus Disease and Activity Index.

## Abbreviations

ACR: American College of Rheumatology
ANA: Antinuclear antibodies
ANOVA: Analysis of variance
CLASI: Cutaneous Lupus Disease Area and Severity Index
DLE: Discoid lupus erythematosus
dsDNA: Double-stranded DNA
ELISA: Enzyme-linked immunosorbent assay
IIF: Indirect immunofluorescence
OD_450_: Optical density at 450 nm
SLEDAI: Systemic Lupus Erythematosus Disease and Activity Index
UTSW: University of Texas Southwestern.
